# Valorization of Recovered Mine Timber as a Secondary Feedstock for Medium-Density Fiberboard Manufacturing

**DOI:** 10.3390/ma18215030

**Published:** 2025-11-04

**Authors:** Viktoria Dudeva, Viktor Savov, Petar Antov, Yuliyan Aleksandrov

**Affiliations:** Department of Mechanical Wood Technology, Faculty of Forest Industry, University of Forestry, 1797 Sofia, Bulgaria; victor_savov@ltu.bg (V.S.);

**Keywords:** recovered wood, mining timber, cascade utilization, medium-density fiberboard, waste valorization, circular bioeconomy

## Abstract

The recovery of timber residues from abandoned underground coal mines presents a unique opportunity to expand the raw material base for wood-based composites, aligning with the principles of cascade utilization. Large amounts of structural wood, embedded for decades under anaerobic and humid mining conditions, remain remarkably well-preserved and can be valorized as a secondary feedstock. The aim of this work was to investigate and evaluate the feasibility of incorporating recovered mining timber into the production of medium-density fiberboards (MDFs). Six types of laboratory panels were produced, containing different ratios of recovered and virgin pine fibers (0–100%), bonded with melamine–urea–formaldehyde resin and hot-pressed at 180 °C. Comprehensive testing of physical and mechanical properties demonstrated that panels with up to 40% recovered fibers fully complied with European standards for general-purpose boards, while up to 60% substitution was acceptable with respect to internal bond strength. At higher substitution levels, however, dimensional stability and strength were significantly reduced. These findings highlight the potential of mine timber recovery as a viable raw material pathway for MDF manufacturing, extending the service life of wood resources and reducing pressure on primary forests. The study emphasizes the role of recovered biomass in advancing circular bioeconomy objectives and resource efficiency in the wood-based panel sector.

## 1. Introduction

Wood-based panels are key engineered materials widely used in construction, furniture manufacturing, and interior design. Among these, medium-density fiberboards (MDFs) have achieved particular prominence due to their uniform density, smooth surfaces, and high machinability, making them suitable for coatings, laminates, and veneering applications [1,2,3,4,5,6]. According to reports from the Food and Agriculture Organization (FAO) and UNECE [6,7,8,9], global consumption of wood-based panels has steadily increased over the past three decades, surpassing 400 million cubic meters in 2019. MDF accounts for approximately 60% of this production volume, confirming its position as the dominant panel type worldwide.

However, the continuing dependence on virgin timber as the primary raw material for MDF manufacturing raises serious environmental concerns. The industry consumes large volumes of industrial roundwood, contributing to deforestation, biodiversity decline, and greenhouse gas emissions. Forecasts by the UNECE suggest that global demand for wood products will increase by 20–30% over the next two decades, further intensifying pressure on forest ecosystems unless alternative lignocellulosic feedstocks are developed [9,10].

In response, international sustainability frameworks, such as the European Green Deal [11] and the EU Circular Economy and Bioeconomy strategies [12], emphasize the transition to renewable, recovered, and recycled wood resources. Within this context, the cascading use principle encourages extending the material life cycle of biomass through multiple sequential uses, i.e., first as a product, then as a recovered raw material, before its final conversion into energy [10,11,12,13,14,15]. These initiatives have accelerated research and industrial interest in the recovery of secondary wood fibers, both from post-consumer waste and from industrial residues, as a means to reduce the sector’s dependence on virgin timber [13,14,15,16].

A growing number of studies demonstrate that recovered fibers can be successfully incorporated into MDF manufacturing without compromising key physical and mechanical properties [17,18,19,20,21]. For example, Xu et al. [17] reported that substitution of up to 25% recovered fibers can maintain comparable board performance, findings consistent with those of Lubis et al. [21]. Halvarsson et al. [16] further confirmed that MDF made from non-wood lignocellulosic materials, such as wheat straw, can achieve adequate strength and dimensional stability. Other studies have shown that process optimization and pretreatments, such as chemical, enzymatic, or hydrothermal, can improve bonding efficiency and fiber quality [18,22,23,24]. Additionally, the integration of agricultural residues and recovered wood fibers has been demonstrated to be technically and economically viable at moderate substitution levels (below 40%) [19,20,21]. Despite this progress, several limitations remain, including increased water absorption, color variation, and reduced internal bond strength at higher recovery ratios [22,23,24].

Recent advances in lignocellulosic biomass valorization have further expanded the potential of recovered wood and secondary fibers as viable raw materials for engineered panels [25,26,27,28,29,30,31,32,33]. Modern research highlights catalytic, biochemical, and thermochemical pathways that enable the transformation of recovered lignocellulosic resources into high-value composites and bio-based polymers [34,35]. Such processes contribute not only to the circular use of biomass but also to reducing carbon emissions and dependence on fossil-derived chemicals. Studies have shown that integrating recovered or modified fibers into composite systems can enhance performance when combined with optimized resin chemistry and surface activation techniques [18,25,26]. Moreover, the development of eco-efficient bio-based adhesives, such as tannin- and lignin-derived binders, further supports the production of environmentally sustainable wood-based panels [36,37]. These trends demonstrate a shift from traditional recycling toward a broader recovery framework that incorporates both material reuse and biochemical valorization within the circular bioeconomy [15,19,34,35,36].

**Figure 1:**
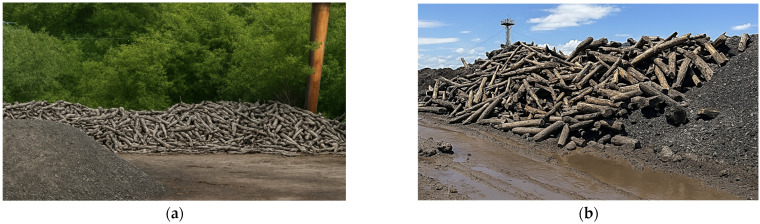
Recovered mining timber from the Pernik coal basin, Bulgaria: (**a**) extracted timber elements; (**b**) detailed view of recovered material.

Bulgaria presents a unique and largely unexplored opportunity for wood recovery through the utilization of timber residues from underground coal mines. Between 1889 and 1997, extensive underground mining activities in the Pernik coal basin consumed millions of cubic meters of timber supports. With the subsequent decline of the coal industry, large volumes of these timbers have remained embedded in abandoned mine galleries. During later open-pit extraction and remediation operations, significant quantities of this long-buried wood have been recovered but are typically historically treated as waste material (Figure 1). The recovery and valorization of this buried timber not only align with circular economy objectives but also introduce an innovative, region-specific source of secondary lignocellulosic feedstock for MDF manufacturing.

Preliminary observations indicate that, despite remaining underground for 65–80 years, much of the recovered timber retains its structural integrity. This durability is attributed to the stable, anaerobic, and humid conditions in the mine galleries. Nevertheless, its chemical composition has likely undergone gradual changes over time, particularly in the relative proportions and structure of lignin, hemicellulose, and cellulose, which may influence its performance as a raw material for composite production.

The present study evaluates the feasibility of recovering coal mine timber and incorporating it into the production of medium-density fiberboards (MDF). Laboratory-scale panels were manufactured with substitution levels ranging from 0% to 100% recovered fibers, and their physical and mechanical properties were systematically evaluated. The results are compared with international findings on the use of secondary lignocellulosic raw materials in MDF manufacturing, allowing the identification of both global trends and region-specific characteristics. Beyond the technical analysis, the study also considers the environmental and economic dimensions of timber recovery in Bulgaria, emphasizing its role in advancing circular economy objectives and improving resource efficiency in the wood-based panel sector.

By pursuing these aims, the study makes several significant contributions. First, it introduces a novel raw material pathway by demonstrating that timber recovered from abandoned underground coal mines can be reincorporated into MDF production at technically viable substitution levels. This resource has been largely neglected in both research and industrial practice, despite millions of cubic meters of such timber remaining embedded in old mining galleries across Europe. Second, the work provides empirical evidence that long-buried mining timber, preserved under anaerobic and humid conditions, retains structural and bonding potential even after decades underground. This challenges prevailing assumptions that such material is irreversibly degraded and unsuitable for high-value applications. Third, the study situates these findings within the broader framework of the circular bioeconomy, showing that recovery of mining timber extends the service life of wood, reduces the demand for virgin forest resources, and diverts substantial volumes of material from disposal or energy recovery.

In this way, the research not only advances the technical understanding of recovered lignocellulosic resources in MDF manufacturing but also highlights their relevance for sustainable industry transformation. By linking material recovery with resource efficiency, climate objectives, and economic competitiveness, the study adds a new dimension to the discourse on circular solutions in the wood-based panel sector.

## 2. Materials and Methods

**Figure 2:**
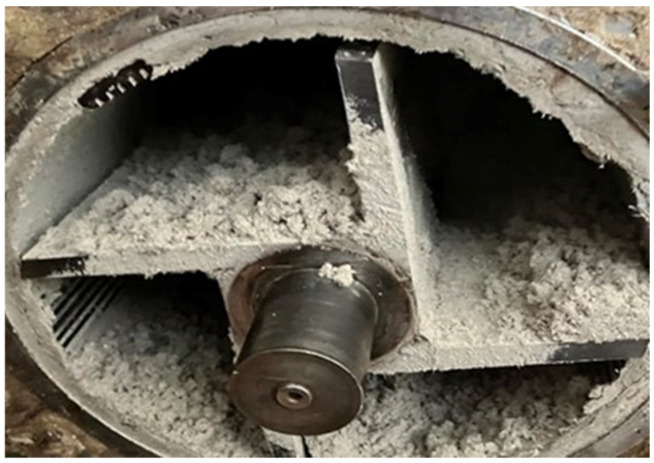
Fiber pulping using a cylindrical laboratory refiner.

Fresh pulp chips derived from Scots pine (*Pinus sylvestris* L.) were supplied by Kronospan Bulgaria EOOD (Veliko Tarnovo, Bulgaria). Timber recovered from abandoned underground coal mines in the Pernik basin, where it had remained embedded for 65–80 years, was used as the secondary raw material. The recovered mining timber consisted of Scots pine (*Pinus sylvestris* L.) support elements. The fresh industrial chips were likewise Scots pine, selected to match the recovered material and ensure comparability between virgin and recovered fibers. The recovered wood, initially employed as structural support elements, typically measured approximately 290 mm in diameter and 980 mm in length. Both the virgin chips and the recovered timber were processed in a cylindrical laboratory refiner (Valmet, Hagfors, Sweden, Figure 2) to obtain fibers suitable for MDF panel production. The initial moisture content of the pulp was 17.2%, which was subsequently reduced to 3.1% using a laboratory kiln.

**Figure 3:**
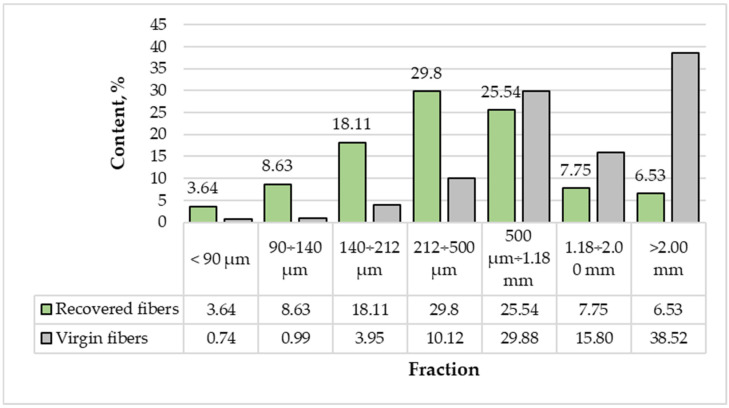
Fractional composition of pulp fibers from recovered and virgin wood.

The fractional composition of the pulp obtained from recovered and virgin wood is presented in Figure 3.

The particle-size distributions of virgin and recovered wood fibers showed significant differences, critical for understanding their physical properties and potential applications in composite materials. Virgin fibers exhibited a broader size range, with the majority of particles concentrated in the coarser fractions. Markedly, 38.52% of virgin fibers were larger than 2 mm, and an additional 15.8% fell within the 1.18–2 mm fraction. In contrast, only 6.53% and 7.75% of recovered fibers were retained in these respective categories. These results indicate that virgin fibers maintain their structural integrity to a greater extent, resulting in a higher proportion of long and coarse particles.

Recovered fibers, in contrast, exhibited a substantial shift toward finer fractions. The highest proportion (29.8%) was within the 212–500 μm range, compared to only 10.12% for virgin fibers. Similarly, 18.11% of recovered fibers were within the 140–212 μm fraction, whereas virgin fibers accounted for only 3.95%. This distribution indicates that long-term underground exposure, combined with the refining process, contributes to fiber shortening and fragmentation, resulting in a markedly higher proportion of fine particles.

Despite these differences, both virgin and recovered fibers demonstrated a notable presence in the 500 μm–1.18 mm fraction, with nearly identical values (29.88% for virgin fibers and 25.54% for recovered fibers). This finding suggests that part of the recovered material retains intermediate-sized fibers, which may still contribute to mechanical reinforcement in composite applications.

In summary, virgin fibers were characterized by a predominance of coarse particles, which enhances mechanical strength and dimensional stability in wood-based composites. Conversely, recovered fibers were dominated by finer fractions, which may improve bonding and surface contact but reduce their overall contribution to mechanical strength.

A melamine–urea–formaldehyde (MUF) resin was selected as the adhesive due to its industrial relevance and compliance with environmental standards. The resin had a solid content of 56% and a pH value of 6.8. The resin dosage was maintained at 10% relative to the oven-dry weight of the fibers, meeting the E1 formaldehyde emission requirements. The use of MUF resin in MDF manufacturing offers several key advantages compared to conventional urea–formaldehyde (UF) adhesives. The partial substitution of urea with melamine significantly improves the hydrolytic stability and water resistance of the cured resin, thereby enhancing the dimensional stability of the resulting panels under humid conditions. In addition, MUF adhesives exhibit improved thermal performance, lower formaldehyde emissions, and greater compatibility with lignocellulosic fibers containing extractives or partially degraded components—an essential factor when using recovered wood [36,37]. These characteristics make MUF resins particularly suitable for applications that target reduced environmental impact and extended service life, aligning with the broader objectives of the circular bioeconomy and sustainable materials development [36,37].

Six medium-density fiberboard (MDF) panel types were produced with recovered-to-virgin fiber ratios of 0:100, 20:80, 40:60, 60:40, 80:20, and 100:0. Given the stability and repeatability of the laboratory pressing setup, one panel (500 mm × 500 mm × 6 mm; target density 850 kg·m^−3^) was produced per formulation. From each panel, ten test specimens per property were prepared and tested (*n* = 10 per property), and results are reported as mean and standard deviation. The production process consisted of fiber preparation, blending with resin, mat forming, cold pressing, and hot pressing.

The blending of fibers with the adhesive was carried out in a laboratory-scale high-speed mixer equipped with needle-shaped blades, operating at a rotational speed of 850 rpm. Resin was applied using a dosing gun with a 1.5 mm nozzle at 0.4 MPa, ensuring uniform resin distribution and consistent fiber coating. Hot pressing was performed at a pressing factor of 15 s·mm^−1^, following a three-stage cycle: 20% of the press time at 2.6 MPa, 30% at 1.5 MPa, and 50% at 0.6 MPa, with a pressing temperature of 180 °C.

After pressing, all panels were conditioned at 20 °C and 65% relative humidity for seven days before testing. Physical and mechanical properties were evaluated according to European standards: EN 323:2001 for density [38], EN 317:1998 for thickness swelling and water absorption [39], EN 310:1999 for bending strength and modulus of elasticity [40], and EN 311:2005 for internal bond strength [41].

For the laboratory production and testing of MDF panels, a universal testing machine (HST WDW 50E, Jinan Hensgrand Instrument Co., Ltd, Jinan, China) was used to determine mechanical properties. Hot pressing was performed using a hydraulic laboratory press (PMC ST 100, Manni S.r.l., Mantua, Italy). Additional equipment included a Kern Dab 100-3 moisture analyzer (KERN & SOHN GmbH, Balingen-Frommern, Germany) for moisture content, a CISA BA 200N sieve shaker (CISA, Barcelona, Spain) for fiber fractionation, and a Kern KB 3600-N2 electronic balance (KERN & SOHN GmbH, Balingen-Frommern, Germany) for precise mass measurements.

For each formulation, one laboratory panel was produced. The ten specimens per property (*n* = 10) are subsamples of the same panel; therefore, between-formulation hypothesis testing on specimen-level data (e.g., ANOVA) was not performed to avoid pseudo-replication. We report mean ± standard deviation and display 95% confidence intervals (CIs) in figures. To quantify monotonic trends across formulations, we fitted weighted linear regressions (WLS) of formulation means versus recovered-fiber ratio (weights = 1/SE^2^, SE = SD/√10), reporting slope estimates with 95% CIs, t statistics (df = 4), and R^2^.

## 3. Results and Discussion

For each panel formulation, differences between variants are assessed descriptively (means with 95% CIs) and via weighted linear regressions of formulation means (*n* = 6) against the recovered-fiber ratio, which provide slope estimates with 95% CIs, t (df = 4), and R^2^ while avoiding pseudo-replication.

**Figure 4:**
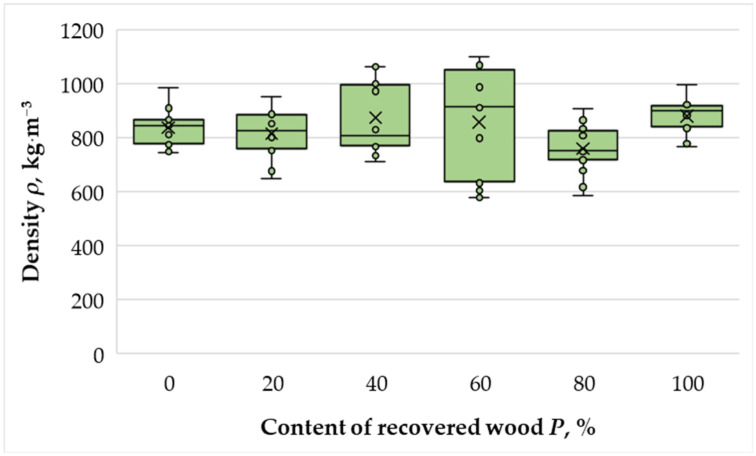
Density of the laboratory-fabricated MDF panels manufactured with different ratios of recovered and virgin wood fibers. Small open circles indicate individual specimen measurements (*n* = 10); the “×” symbol denotes the mean; the box represents the interquartile range (Q1–Q3) with the horizontal line at the median; whiskers span the minimum–maximum values.

The density of manufactured panels is presented in Figure 4. 

Due to the use of mechanical stops during hot pressing, the density of the laboratory-produced panels varied within a relatively narrow range of 809–869 kg·m^−3^, which was close to the target density of 850 kg·m^−3^. According to the requirements of EN 323:2001 [38], such variation falls within the typical range for MDF panels. The overall coefficient of variation was 7.42%, indicating good process consistency and confirming the reliability of laboratory-scale pressing conditions.

A uniform density profile across all experimental series is crucial for ensuring comparable mechanical and physical properties, since density strongly affects parameters such as modulus of rupture (MOR), internal bond strength (IB), and dimensional stability. The results indicated that density differences among the samples were minor and were therefore not expected to significantly influence the comparative evaluation of panel performance. The obtained values were consistent with those reported for industrial MDF panels of similar nominal density (830–870 kg·m^−3^) [2], confirming the adequacy of the applied hot-pressing schedule and adhesive distribution.

**Figure 5:**
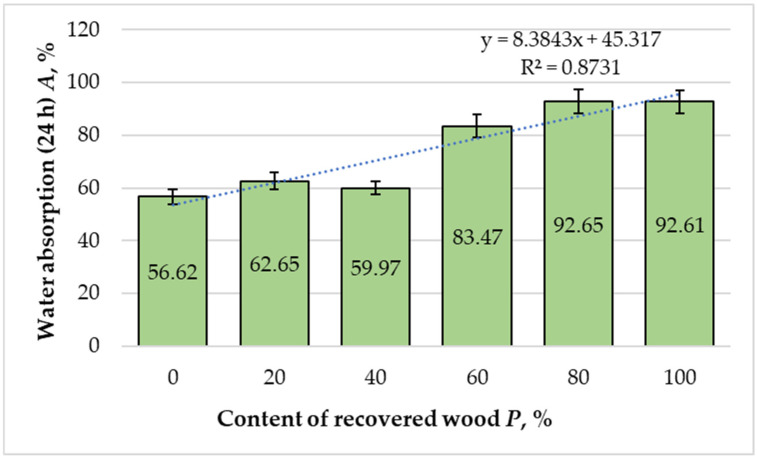
Variation in water absorption of the laboratory-fabricated MDF panels manufactured with different ratios of recovered and virgin wood fibers. Bars show the mean (*n* = 10) and error bars indicate the 95% confidence interval (CI = mean ± 2.262·SD/√10). Trends across formulations were assessed by weighted linear regression on formulation means.

Figure 5 presents the variations in water absorption (WA) of the MDF panels as a function of the recovered fiber content. A significant positive trend with the recovered-fiber ratio was observed (slope = +0.398% per 1%, 95% CI 0.137 to 0.659, t = 4.24, df = 4, R^2^ = 0.873), i.e., ≈ +3.98% WA for each 10% increase in recovered fibers.

Panels manufactured exclusively from virgin fibers exhibited a WA value of 56.62%. With the addition of 20% recovered fibers, WA increased to 62.65%, corresponding to a 9.62% rise relative to the reference panel. Interestingly, at a 40% substitution level, a slight reduction to 59.97% was observed, indicating that intermediate recovery ratios may promote a denser fiber network and more efficient resin distribution, partially compensating for the higher porosity of the recycled fibers. However, beyond this threshold, the influence of the recovered material became dominant: at 60% substitution, WA increased sharply to 83.47%, and for panels containing 80% and 100% recovered fibers, values reached 92.65% and 92.61%, respectively.

Regression analysis (R^2^ = 0.8731) confirmed a strong positive correlation between recovered fiber content and WA values, suggesting that the substitution ratio exerts a direct and significant effect on hygroscopic behavior. The increase in water uptake can be attributed to several interrelated factors. Recovered fibers generally exhibit a higher degree of morphological degradation, including broken lumens, fissured cell walls, and fibrillation, which increases the specific surface area and enhances capillary absorption [15,16,17,18,19,22,23]. Additionally, the partial loss of hemicellulose and amorphous cellulose during long-term exposure or recovery processes increases the availability of hydrophilic sites, further facilitating moisture adsorption [18,19].

Another contributing factor is the reduced resin coverage in high recovery ratios, as finer and more heterogeneous fibers hinder uniform adhesive distribution. This leads to localized zones of insufficient bonding, creating open capillaries that facilitate water penetration [22,23]. Similar findings have been reported in studies of MDF panels incorporating recycled and agro-based fibers, where excessive substitution levels resulted in impaired resin-fiber interaction and increased thickness swelling [15,16,17,18,19,22].

Overall, these results confirmed that although moderate incorporation of recovered fibers may not drastically compromise water resistance, higher substitution ratios significantly increase hygroscopicity and moisture sensitivity. Therefore, process optimization, particularly through resin modification, fiber pretreatment, or surface densification, is essential for improving the dimensional stability of MDF panels produced from recovered lignocellulosic materials.

**Figure 6:**
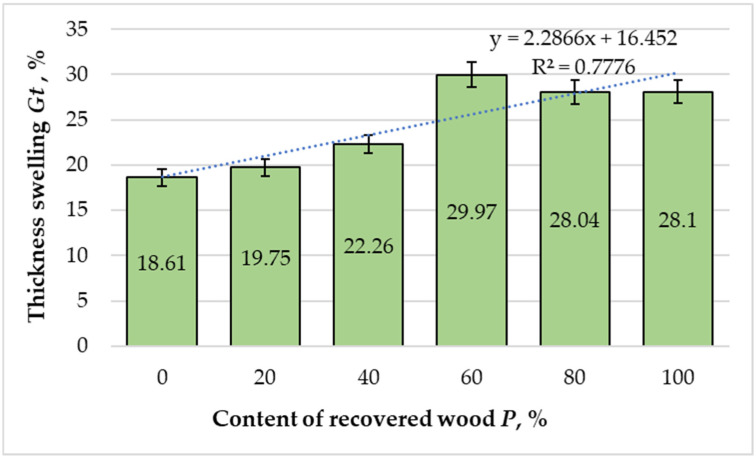
Variation in thickness swelling of the laboratory-fabricated MDF panels manufactured with different ratios of recovered and virgin wood fibers. Bars show the mean (*n* = 10) and error bars indicate the 95% confidence interval (CI = mean ± 2.262·SD/√10). Trends across formulations were assessed by weighted linear regression on formulation means.

Figure 6 presents the variations in thickness swelling (TS) of the MDF panels as a function of the recovered fiber content. TS increased significantly with recovered content (slope = +0.115% per 1%, 95% CI 0.044 to 0.185, t = 4.53, df = 4, R^2^ = 0.778), ≈ +1.15% per 10%.

The reference panel, produced from 100% virgin fibers, exhibited a TS value of 18.61% after 24 h of water immersion. With 20% recovered fibers, swelling increased modestly to 19.75%, while at 40% substitution it rose further to 22.26%. The most pronounced expansion occurred at 60% recovered fiber content, reaching 29.97%. Markedly, at higher substitution levels (80% and 100%), a slight reduction was observed, at 28.04% and 28.10%, respectively. These variations suggest that the relationship between recovered fiber proportion and dimensional stability is not strictly linear but is influenced by fiber morphology, resin distribution, and inter-fiber packing density.

The regression model (R^2^ = 0.7776) revealed a moderate to strong positive correlation between recovered fiber content and TS values. The general tendency for increased swelling with higher recovery ratios can be attributed to the reduced inter-fiber bonding strength and compromised cell wall integrity of recovered fibers [16,17,22]. These fibers exhibit structural discontinuities, open lumens, and microfissures, which facilitate the penetration of water molecules and promote volumetric expansion upon moisture uptake [23,24]. Furthermore, finer, more heterogeneous fiber fractions typically reduce resin wetting and penetration efficiency, resulting in weaker adhesive bridges within the fiber network [17,22]. This phenomenon was also reported by Lykidis and Grigoriou [24], who observed similar increases in swelling for MDF produced from hydrothermally recycled fibers.

The observed decline in TS beyond the 60% substitution level may result from densification effects and partial compression of the fiber mat during hot pressing, which can mechanically limit further expansion. However, this apparent stabilization is likely superficial, as internal stresses and microcracks may still exist within the matrix, potentially affecting long-term dimensional stability.

Despite the overall increasing trend, all panels remained below the maximum threshold value of 30% specified by EN 622-5 [42] for load-bearing MDF panels under both dry and humid conditions. These findings demonstrate that, although high recovery ratios reduce water resistance and dimensional stability, MDF containing up to 60% recovered fibers can still meet the standard performance criteria. This highlights the potential for partial substitution of virgin fibers with recovered material, provided that process optimization, especially resin formulation and fiber pretreatment, is employed to mitigate swelling effects.

**Figure 7:**
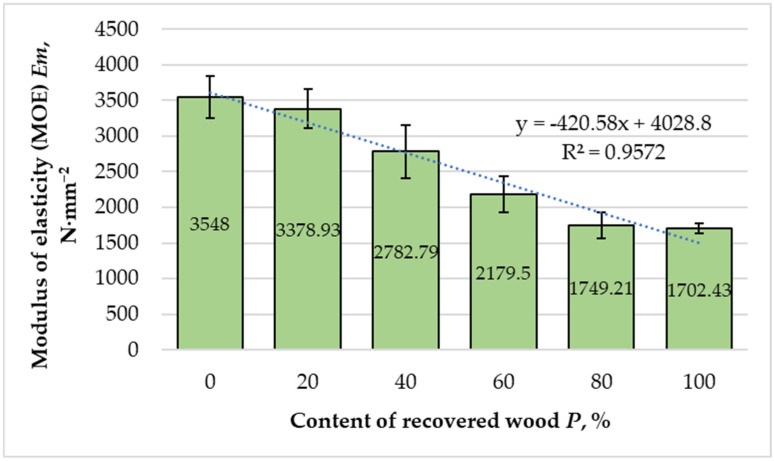
Variation in modulus of elasticity of the laboratory-fabricated MDF panels manufactured with different ratios of recovered and virgin wood fibers. Bars show the mean (*n* = 10) and error bars indicate the 95% confidence interval (CI = mean ± 2.262·SD/√10). Trends across formulations were assessed by weighted linear regression on formulation means.

The variation in the modulus of elasticity (MOE) with increasing recovered fiber content is shown in Figure 7. MOE showed a strong negative trend (slope = −18.47 N·mm^−2^ per 1%, 95% CI −24.81 to −12.13, t = −8.09, df = 4, R^2^ = 0.957), ≈ −185 N·mm^−2^ per 10%.

Panels manufactured entirely from virgin fibers exhibited the highest MOE value of 3548 N·mm^−2^, serving as the reference benchmark. With a 20% substitution of recovered fibers, the modulus decreased slightly to 3378.93 N·mm^−2^, corresponding to a reduction of 4.65%. At higher recovery ratios, the decline became progressively more pronounced: 2782.79 N·mm^−2^ at 40%, 2179.50 N·mm^−2^ at 60%, 1749.21 N·mm^−2^ at 80%, and 1702.43 N·mm^−2^ for panels composed entirely of recovered fibers. Overall, this represents a 52% reduction relative to the reference value, clearly indicating the adverse effect of high recovery content on panel stiffness.

Regression analysis (R^2^ = 0.9572) revealed a strong and nearly linear negative correlation between recovered fiber content and MOE. This behavior can be attributed primarily to the deteriorated microstructure of recovered fibers, which exhibit reduced aspect ratios, weakened cell walls, and diminished intrinsic stiffness [16,19,22]. Shorter fibers transfer stresses less effectively within the adhesive matrix, resulting in lower load-bearing efficiency and stiffness. Additionally, the higher proportion of fines typical of recovered materials increases resin demand and reduces the effectiveness of inter-fiber bonding, thereby limiting stress-transfer capacity [23,24]. Consequently, the composite response becomes more matrix-dominated, with elasticity determined mainly by the resin phase rather than the fiber framework.

The observed decline in stiffness aligns with earlier findings by Halvarsson et al. [16] and Lykidis and Grigoriou [24], who reported that recycled or hydrothermally treated fibers exhibit inferior bonding and mechanical performance compared with virgin counterparts. According to EN 622-5 [42], panels containing up to 40% recovered fibers meet the minimum MOE requirement for general-purpose MDF used in humid conditions (≥2700 N·mm^−2^). Furthermore, those incorporating up to 20% recovered fibers also meet the stricter requirement for load-bearing panels in dry conditions (≥3000 N·mm^−2^). Beyond this threshold, however, stiffness decreases below standard limits, confirming that excessive recovery ratios compromise structural integrity.

From a materials engineering perspective, the reduction in MOE with increasing recovery ratio highlights the need to optimize both fiber morphology and interfacial adhesion. Strategies such as fiber surface activation, coupling-agent modification, or the use of high-performance MUF resins could mitigate stiffness losses by enhancing fiber–matrix bonding. Therefore, while recovered fibers can effectively replace a portion of virgin raw material, maintaining mechanical performance at elevated substitution levels demands tailored bonding chemistry and precise control of manufacturing parameters.

**Figure 8:**
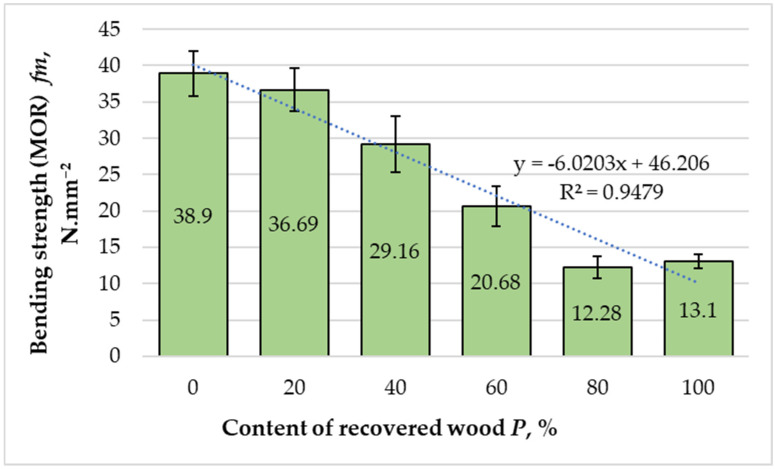
Variation in bending strength of the laboratory-fabricated MDF panels manufactured with different ratios of recovered and virgin wood fibers. Bars show the mean (*n* = 10) and error bars indicate the 95% confidence interval (CI = mean ± 2.262·SD/√10). Trends across formulations were assessed by weighted linear regression on formulation means.

The variation in bending strength (MOR) with increasing recovered fiber content is presented in Figure 8. MOR decreased markedly with recovered content (slope = −0.307 N·mm^−2^ per 1%, 95% CI −0.390 to −0.224, t = −10.25, df = 4, R^2^ = 0.948), ≈ −3.07 N·mm^−2^ per 10%.

The reference panel composed entirely of virgin fibers achieved the highest MOR value of 38.9 N·mm^−2^. With the incorporation of 20% recovered fibers, bending strength decreased moderately to 36.69 N·mm^−2^, corresponding to a 5.68% reduction. At a 40% substitution, the decline became more pronounced (29.16 N·mm^−2^), while at a 60% recovery, the strength dropped to 20.68 N·mm^−2^. For panels containing 80% and 100% recovered fibers, the MOR values were 12.28 N·mm^−2^ and 13.10 N·mm^−2^, respectively. Overall, this represents a 73% reduction compared with the reference panel, indicating a substantial loss in bending performance with increasing recovery ratio.

Regression analysis (R^2^ = 0.9479) confirmed a strong linear correlation between recovered fiber content and the decline in bending strength. The reduction in MOR can be attributed to the microstructural degradation of recovered fibers, including shortened fiber length, fractured cell walls, and reduced stiffness, which collectively weaken stress transfer within the composite matrix [16,19]. The heterogeneous nature of recovered fiber mats also promotes non-uniform stress distribution during bending, leading to premature interlaminar failure and crack initiation. Furthermore, incomplete resin penetration and poor adhesive wetting in the porous recovered fiber network result in reduced interfacial adhesion and lower bond integrity, increasing the likelihood of brittle fracture under bending loads [23,24]. 

The decreasing MOR trend closely parallels the decline in MOE values, confirming that both stiffness and strength are primarily governed by the quality of fiber bonding and the efficiency of load transfer mechanisms. According to EN 622-5 [42], MDF panels containing up to 40% recovered fibers meet the minimum bending strength requirement for general-purpose boards in humid environments (≥27 N·mm^−2^). Panels with up to 20% recovered fibers also comply with the stricter specifications for load-bearing applications (≥30 N·mm^−2^). Above 40% substitution, however, the MOR values fall below these thresholds, indicating that excessive recovery ratios compromise the material’s structural integrity.

The pronounced, nearly linear decrease in both MOE and MOR (R^2^ > 0.94) highlights the critical roles of fiber morphology and interfacial bonding in determining the mechanical performance of MDF. The observed behavior suggests a threshold around 40% recovered fiber content, beyond which mechanical integrity deteriorates sharply. Below this limit, partial recovery appears technically feasible, allowing for meaningful reductions in virgin raw material use without substantial loss of performance. However, higher substitution ratios would require improvements in adhesive formulation, fiber surface activation, or hot-pressing optimization to restore interfacial bonding and mitigate strength loss. We note that our conclusions regarding fiber microstructural damage (e.g., lumen collapse, cell-wall fissures, fines morphology) are inferred from fractionation trends and prior literature. Recognizing the importance of direct microscopic evidence and the space limitations of a single article, a dedicated LM/SEM study comparing virgin and recovered fibers is planned as follow-up work to provide visual and quantitative confirmation. Comprehensive compositional (chemical) analyses of virgin vs. recovered fibers (extractives, carbohydrate fractions, lignin) and surface-chemistry assays (FTIR, XPS) will also be performed to better relate fiber chemistry to interfacial bonding and hygroscopic behavior.

**Figure 9:**
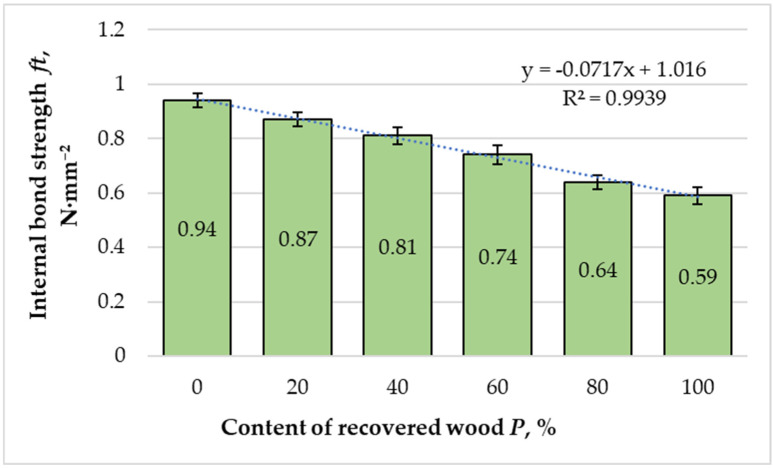
Variation in internal bond strength of the laboratory-fabricated MDF panels manufactured with different ratios of recovered and virgin wood fibers. Bars show the mean (*n* = 10) and error bars indicate the 95% confidence interval (CI = mean ± 2.262·SD/√10). Trends across formulations were assessed by weighted linear regression on formulation means.

The variation in internal bond (IB) strength as a function of recovered fiber content is presented in Figure 9. IB declined strongly (slope = −0.00361 N·mm^−2^ per 1%, 95% CI −0.00403 to −0.00318, t = −26.87, df = 4, R^2^ = 0.994), ≈ −0.036 N·mm^−2^ per 10%.

The reference panel composed entirely of virgin fibers exhibited the highest IB strength value of 0.94 N·mm^−2^. With increasing proportions of recovered fibers, a gradual but consistent decline in IB strength was observed: 0.87 N·mm^−2^ at 20%, 0.81 N·mm^−2^ at 40%, 0.74 N·mm^−2^ at 60%, 0.64 N·mm^−2^ at 80%, and 0.59 N·mm^−2^ for panels composed entirely of recovered fibers. The total reduction relative to the reference value was approximately 37%, indicating a substantial loss in inter-fiber bonding efficiency as the proportion of recovered material increased.

Regression analysis (R^2^ = 0.9939) revealed a robust linear relationship between recovered fiber content and internal bond strength, indicating that fiber quality degradation directly influences interfacial adhesion. The weakening effect can be attributed to several factors. Recovered fibers are typically shorter, more fragmented, and exhibit microstructural damage such as collapsed cell lumens and delaminated walls, which reduce their effective surface area for resin anchoring [15,16,22,23]. Additionally, thermal and biological aging during long-term underground exposure may alter surface chemistry and reduce the availability of hydroxyl groups, further limiting resin–fiber interaction. These factors result in fewer and weaker adhesive bridges within the fiber network, decreasing internal cohesion and resistance to delamination.

From a structural performance perspective, IB strength is one of the most sensitive indicators of adhesive efficiency in MDF. Reduced IB values indicate incomplete resin penetration and non-uniform adhesive distribution in fiber mats that contain a high proportion of fines and degraded fibers. Similar trends have been reported in studies on the hydrothermal and mechanical recovery of MDF fibers, where fiber damage led to weaker bonding and premature interfacial failure [22,23].

According to EN 622-5 [42], the minimum IB strength required for load-bearing MDF panels in humid conditions is 0.65 N·mm^−2^. Panels containing up to 60% recovered fibers met this requirement, demonstrating acceptable inter-fiber adhesion and mechanical integrity. However, panels with 80% and 100% recovered fibers failed to reach the standard threshold, confirming that excessive substitution compromises cohesive strength.

Overall, the IB results confirm the progressive mechanical deterioration observed in MOE and MOR values. The pronounced linear dependency between recovery ratio and bond strength indicates that mechanical performance is primarily controlled by fiber morphology and adhesive efficiency. Despite this decline, panels incorporating up to 60% recovered fibers maintained sufficient internal cohesion for practical applications, representing a technically viable substitution level that balances material recovery with performance. Beyond this threshold, additional surface modification or resin optimization strategies, such as increasing the melamine content or using coupling agents, would be necessary to restore bond quality and maintain compliance with industrial standards.

The experimental results revealed a consistent pattern across all measured properties, demonstrating the significant effect of recovered fiber content on MDF panel performance.

Regarding hygroscopic behavior, both WA and TS increased with higher proportions of recovered fibers. This trend reflects the finer fraction, greater specific surface area, and microstructural damage characteristic of recovered material, which collectively enhance hygroscopicity and capillary water uptake. Nevertheless, all panels remained within the EN 622-5 [42] threshold of 30% thickness swelling, demonstrating that dimensional stability requirements can still be met even at 100% substitution when appropriate pressing parameters and resin systems are applied. These findings suggest that controlled manufacturing conditions—specifically, resin distribution, pressing temperature, and densification—can partially mitigate the inherently higher porosity of recovered fibers.

For mechanical properties, a strong negative relationship was observed between the recovered fiber content and both MOE and MOR values. The decline in stiffness and bending strength is primarily due to reduced fiber length, lower intrinsic stiffness, and decreased efficiency of stress transfer across the fiber–resin matrix. Panels containing up to 40% recovered fibers fulfilled the EN 622-5 [42] requirements for general-purpose boards in humid environments, while those with up to 20% substitution also satisfied the stricter criteria for load-bearing applications. Beyond this threshold, however, the structural limitations of the recycled fiber network become dominant, leading to a substantial deterioration of mechanical integrity.

A comparable trend was identified for IB strength. Although IB values declined progressively with increasing recovered fiber content, panels with up to 60% substitution still met the standard limit of 0.65 N·mm^−2^ for load-bearing boards in humid conditions, as specified in EN 622-5 [42]. The observed reduction in cohesion can be attributed to the weaker inter-fiber adhesion and limited formation of resin bridges resulting from surface degradation and chemical aging of the recovered fibers [19,23,24]. At substitution ratios exceeding 60%, insufficient adhesion between the fiber network and the adhesive matrix resulted in interlaminar failure and internal delamination under load.

Overall, incorporating recovered fibers into MDF is technically feasible up to 40–60%, depending on the property considered. Within this range, the panels meet relevant European standards and exhibit performance comparable to conventional MDF, while significantly reducing the use of virgin timber. Beyond these limits, the sharp decrease in mechanical properties outweighs the environmental and economic benefits of higher substitution. Nonetheless, partial recovery represents a viable strategy for sustainable production, balancing resource efficiency, waste reduction, and material performance.

The results of this study are consistent with previously published research. Xu et al. [17] demonstrated that incorporating up to 25% recovered fibers maintains standard compliance. At the same time, Zeng, Q. et al. [18] confirmed the technical feasibility of 40% substitution without significant losses in strength or stiffness. Neitzel et al. [19] and Nguyen et al. [23] emphasized that fiber morphology and adhesive interactions are key parameters that determine performance in recovered-fiber composites. Lykidis and Grigoriou [24] highlighted hydrothermal treatment as a practical pre-conditioning step to restore bonding potential, and Roffael [30,31,32,33] reported that fiber–adhesive chemistry plays a decisive role in achieving sufficient interfacial cohesion in recycled wood composites.

By validating that MDF panels containing up to 40–60% recovered fibers comply with EN 622-5 [42] requirements across multiple properties, this study extends current knowledge on the utilization of secondary lignocellulosic materials. Most importantly, it provides the first experimental evidence of the feasibility of producing MDF panels from long-buried coal mine timber—a source previously considered unsuitable for industrial reuse. This finding not only contributes to advancing sustainable material recovery but also demonstrates a novel pathway for implementing the circular economy within the European wood-based panel sector. The successful recovery and valorization of this unique lignocellulosic resource could significantly enhance raw material efficiency and reduce the carbon footprint of panel manufacturing, positioning recovered timber as a promising component in next-generation bio-based composite materials.

From a technological standpoint, the results reveal clear potential for advancing the use of recovered fibers beyond the identified threshold through targeted process innovations. Optimizing the adhesive content and distribution, combined with chemical surface activation or hydrophobization of fibers, could compensate for the morphological degradation of the recovered material. Future developments in hybrid melamine–urea–formaldehyde systems, the incorporation of nanoreinforcements, or the application of coupling agents may further enhance fiber–resin compatibility, enabling high-performance MDF panels with greater proportions of recovered timber. Moreover, the study establishes a foundation for process scaling under industrial conditions, providing quantitative benchmarks for mechanical and physical performance.

From an industrial perspective, the identified optimum substitution range of 40–60% recovered fibers represents a practical balance between resource recovery and performance retention. Within this interval, the manufactured panels complied with EN 622-5 requirements for dimensional stability and mechanical strength, confirming their suitability for general-purpose and load-bearing applications. This substitution level corresponds to a potential reduction in virgin fiber consumption by 0.4–0.6 m^3^ per m^3^ of MDF, directly lowering the demand for primary timber resources. Assuming that virgin fiber MDF production is associated with approximately 470–600 kg CO_2_-eq per m^3^ of panel (cradle-to-gate, fossil emissions) [29,43,44], a 40–60% substitution with recovered fibers could yield estimated carbon savings of 190–360 kg CO_2_-eq per m^3^, while simultaneously preventing the uncontrolled degradation of recovered mine timber and its associated greenhouse-gas release. The valorization of mine timber thus provides a dual environmental benefit: extending the service life of existing biomass and reducing the sector’s raw material footprint in alignment with EU Circular Economy and Green Deal objectives. In practical terms, recovered mine timber can be integrated into existing fiber preparation lines with minor adjustments to refining and blending parameters, offering a cost-effective pathway toward more sustainable and resource-efficient MDF production.

From an environmental and circular economy perspective, this research presents a model example of waste valorization through material recovery. The reintegration of coal mine timber into MDF production directly supports the objectives of the European Union’s Circular Economy Action Plan by extending the life cycle of wood resources, reducing the need for virgin timber, and minimizing waste disposal. This approach not only prevents the environmental burden associated with decomposing buried wood but also enhances overall resource efficiency within the wood-based panel industry. By transforming a previously neglected mining byproduct into a functional raw material, the study bridges the gap between ecological responsibility and material innovation.

Economically, the valorization of mining timber residues offers a cost-effective and locally available raw material alternative in regions where access to high-quality virgin timber is limited. Partial substitution of fresh fibers with recovered ones could stabilize production costs, strengthen raw material security, and improve the competitiveness of local MDF manufacturers in both domestic and export markets. Furthermore, this strategy aligns with broader European sustainability policies by promoting the regional utilization of resources and reducing dependency on imported raw materials.

## 4. Conclusions

This study provides compelling experimental evidence that long-buried coal mine timber can be successfully recovered and reincorporated into medium-density fiberboard (MDF) production. The results demonstrate that substitution levels of up to 40–60% recovered fibers can be achieved without violating the requirements of EN 622-5 for dimensional stability and mechanical performance. Within this range, the panels exhibited acceptable water absorption, thickness swelling, bending strength, modulus of elasticity, and internal bond strength, confirming the technical feasibility of partial replacement of virgin fibers. At higher substitution ratios, however, the pronounced increase in hygroscopic behavior and the deterioration of mechanical properties highlighted the structural constraints inherent to highly recycled fiber networks.

The primary novelty and scientific contribution of this research lie in the demonstration of the recovery and reuse of long-buried coal mine timber—a unique lignocellulosic material preserved for 65–80 years under anaerobic, humid, and mineral-rich conditions. Unlike prior work mainly addressing short-lived industrial residues or furniture waste, this study introduces a previously untapped category of recovered wood and provides quantitative evidence of its suitability for MDF manufacturing at technically viable substitution levels.

Beyond the technical feasibility, the use of 40–60% recovered mine timber in MDF manufacturing carries substantial environmental and economic relevance. At this substitution level, virgin timber demand can be reduced by nearly half, translating into lower embodied carbon, improved waste valorization, and regional circularity benefits. The integration of this secondary feedstock aligns with EU sustainability priorities, offering a replicable model for the low-carbon transformation of the wood-based panel industry.

Looking ahead, there is clear potential to advance performance beyond the identified threshold through targeted process innovations. Optimizing adhesive content and distribution and exploring coupling agents and fiber hydrophobization may compensate for the fines-rich, morphologically degraded fraction of recovered fibers, thereby improving interfacial bonding. To strengthen structure–property relationships, we will conduct direct microscopic analyses (LM/SEM) of virgin versus recovered fibers, complemented by surface and compositional assays (FTIR/XPS and determination of extractives, polysaccharide fractions, and lignin). In parallel, long-term durability under cyclic humidity and temperature, industrial-scale validation, and streamlined life-cycle assessment will be pursued to quantify performance robustness and sustainability benefits. Taken together, these steps aim to refine the manufacturing window and, where feasible, extend the viable substitution level beyond 60% while maintaining compliance with EN 622-5.

In summary, the work establishes a scientifically and industrially relevant pathway to valorize long-buried mine timber in MDF production. While challenges persist at high substitution levels, the demonstrated feasibility within the 40–60% range offers a practical means to reduce reliance on virgin fibers and to integrate a neglected resource into circular, resource-efficient panel manufacturing.

## Data Availability

The data presented in this study are available on request from the corresponding authors.
